# Microtubule cross-linking activity of She1 ensures spindle stability for spindle positioning

**DOI:** 10.1083/jcb.201701094

**Published:** 2017-09-04

**Authors:** Yili Zhu, Xiaojing An, Alexis Tomaszewski, Peter K. Hepler, Wei-Lih Lee

**Affiliations:** 1 Molecular and Cellular Biology Graduate Program, University of Massachusetts, Amherst, MA; 2 Biology Department, University of Massachusetts, Amherst, MA

## Abstract

Dynein mediates spindle positioning in budding yeast by pulling on astral microtubules (MTs) from the cell cortex. The MT-associated protein She1 regulates dynein activity along astral MTs and directs spindle movements toward the bud cell. In addition to localizing to astral MTs, She1 also targets to the spindle, but its role on the spindle remains unknown. Using function-separating alleles, live-cell spindle assays, and in vitro biochemical analyses, we show that She1 is required for the maintenance of metaphase spindle stability. She1 binds and cross-links MTs via a C-terminal MT-binding site. She1 can also self-assemble into ring-shaped oligomers. In cells, She1 stabilizes interpolar MTs, preventing spindle deformations during movement, and we show that this activity is regulated by Ipl1/Aurora B phosphorylation during cell cycle progression. Our data reveal how She1 ensures spindle integrity during spindle movement across the bud neck and suggest a potential link between regulation of spindle integrity and dynein pathway activity.

## Abstract (precis)

Dynein orients the spindle by pulling on astral microtubules from the cortex. In *Saccharomyces cerevisiae*, the microtubule-associated protein She1 specifically inhibits dynein in the mother compartment to promote spindle movements toward the bud. Zhu et al. demonstrate that She1 also stabilizes interpolar microtubules, ensuring spindle integrity during dynein-mediated spindle positioning.

## Introduction

Mechanical stability of the spindle is important for various mitotic processes and is particularly critical for cell divisions requiring the spindle to move or orientate to a specific location or cell division axis. Many examples of spindle movements occur during metaphase, including spindle rotation in *Drosophila melanogaster* neuroblasts (Kaltschmidt et al., 2000), spindle centering in mammalian cultured cells (Collins et al., 2012; Kiyomitsu and Cheeseman, 2012), and spindle (or nuclear) positioning in fungal hyphae (Gibeaux et al., 2017). Proper stability of the spindle is achieved through a balance of opposing forces generated by motor and nonmotor microtubule (MT)-associated proteins (MAPs) located at various sites within the spindle and/or cell cortex (Dumont and Mitchison, 2009; Goshima and Scholey, 2010). In particular, MT cross-linkers that hold the spindle in place can act as passive force resistors (Syrovatkina et al., 2013), resisting the pulling forces exerted on astral MTs during spindle movements. These resistive forces could prevent spindle deformation, ensuring spindle integrity during positioning events, and may operate at the kinetochore and/or the antiparallel MT overlap in the spindle center.

Budding yeast provides an ideal system to dissect the mechanism required for the maintenance of spindle stability during spindle positioning. Its metaphase spindle is relatively small and simple (Winey and O’Toole, 2001; Winey and Bloom, 2012), with 32 kinetochore MTs and ~8 interpolar MTs (ipMTs). Once formed, the metaphase spindle maintains a characteristic length of ~2 µm for ~15–20 min before anaphase, during which it remains fairly rigid and can rotate up to 90° to align along the mother bud axis (Winey and Bloom, 2012). A classic genetic study suggests that the metaphase spindle is stabilized by counteracting forces produced by the kinesin-5 motors Cin8 and Kip1 and the kinesin-14 motor Kar3 (Saunders and Hoyt, 1992). More recent data indicate that additional passive cross-linking proteins are required to maintain proper MT bundling for stable spindle bipolarity during metaphase (Hepperla et al., 2014). However, the proteins involved have not been ascribed, and how their activities might be regulated during cell cycle progression is unknown.

To achieve faithful partitioning of duplicated chromosomes, the yeast spindle must pass through a small bud neck (<2 µm wide) between the mother and daughter cell. Dynein moves the metaphase spindle into the neck by pulling on astral MTs from sites at the cell cortex (Adames and Cooper, 2000; Moore et al., 2009b). Remarkably, in *kar9Δ* cells, the preanaphase (metaphase-like) spindle could withstand dynein-dependent pulling forces that cause it to oscillate back and forth across the bud neck (Yeh et al., 2000; Moore et al., 2009a). These observations suggest that an active mechanism may operate in yeast to maintain spindle integrity against the dynein pulling force, but how such a mechanism might coordinate with dynein activity during spindle translocation is unclear. The dynein pathway in *Saccharomyces cerevisiae* (Markus et al., 2012a; Cianfrocco et al., 2015) includes components of the dynein and dynactin complexes, the cortical attachment molecule Num1, and regulators that localize to the MT plus ends (Pac1/LIS1, Ndl1/NudEL, Bik1/Clip170, and Kip2) and along the MT lattice (She1). Of these, only She1 localizes to the mitotic spindle (Wong et al., 2007; Woodruff et al., 2009; Pigula et al., 2014).

Here, we have combined genetic and cell biological assays with biochemical and biophysical characterizations to dissect the role of She1 on the spindle MTs. Because *SHE1* deletion leads to hyperactivity of cortical dynein (Woodruff et al., 2009; Bergman et al., 2012), which could potentially obscure the analysis of its role on the spindle, we first generated truncation alleles to map the domain required for dynein-regulating activity. Subsequent analysis of the alleles that retained dynein-regulating function revealed an unexpected role for She1 in the maintenance of spindle integrity. We found that She1 stabilizes ipMTs and that this activity is regulated by Ipl1/Aurora B phosphorylation. Our data reveal how She1 coordinates spindle integrity with spindle positioning across the bud neck and offer insight into how metaphase spindles could be stabilized by a passive cross-linking MAP during dynein-mediated spindle movements.

## Results

### Mapping She1’s domain required for dynein-regulating activity

**Figure 1.**
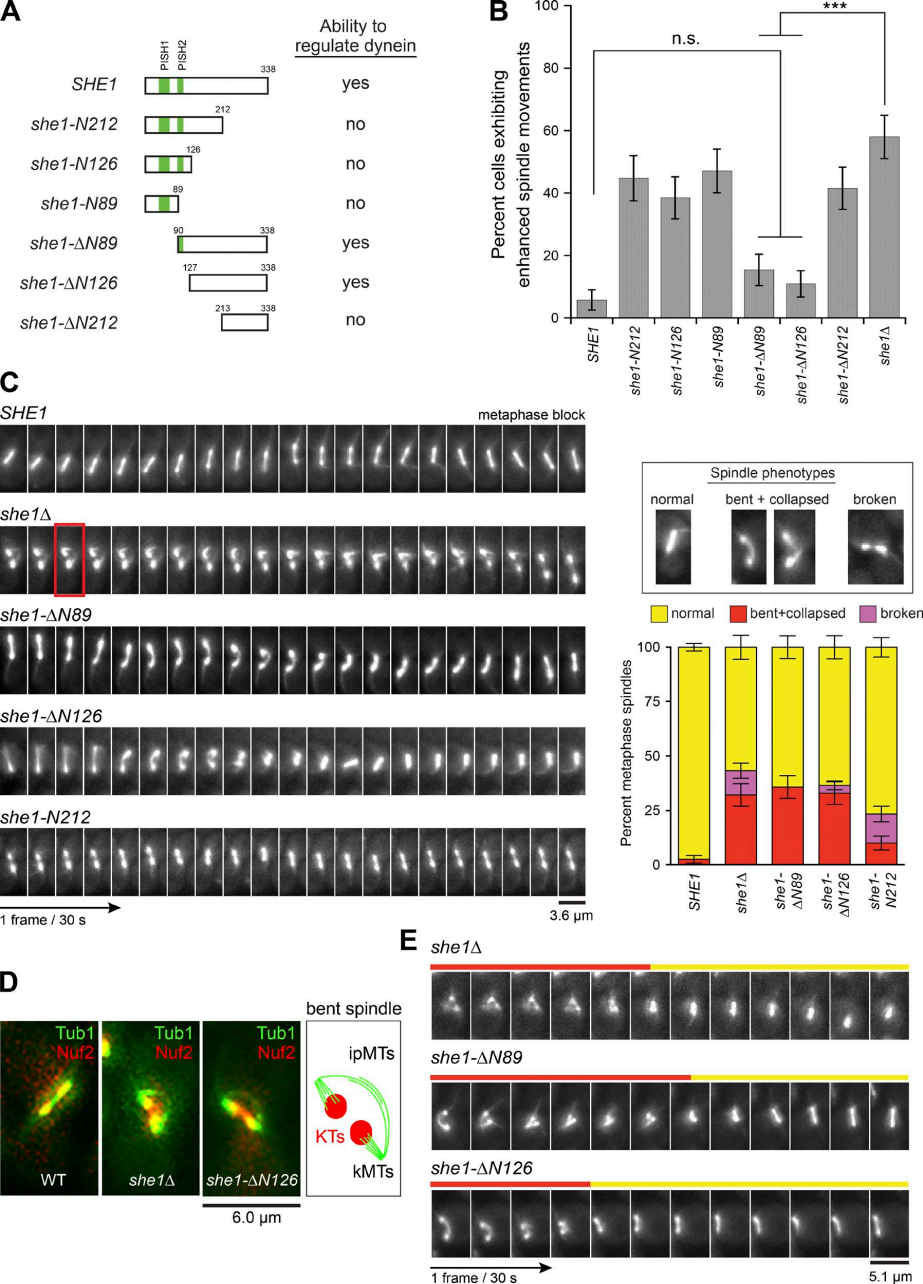
**She1 is required for the maintenance of metaphase spindle stability.** (A) Diagram of genomic *she1* alleles and their ability to regulate cortical dynein activity. (B) Frequency of observing cells with enhanced dynein activity in each indicated strain (*n* ≥ 47 cells). n.s., not statistically significant, P > 0.1; ***, P < 0.0001 by unpaired *t* test. (C) Video frames showing spindle defects during metaphase arrest in *MET3-CDC20 GFP-TUB1* cells. Frame boxed in red is shown in merge with Nuf2-mCherry in D. Right, graph showing frequency of observing indicated spindle phenotypes for each strain (*n* ≥ 80 cells each). Error bars in B and C are standard error of proportion. (D) Bending of ipMTs in metaphase-arrested WT, *she1Δ*, and *she1-ΔN126* cells. KT, kinetochore; kMT, kinetochore MT. (E) Video frames showing recovery of a bent/collapsed spindle in *she1Δ*, *she1-ΔN89*, and *she1-ΔN126* cells. Red or yellow indicates bent/collapsed or normal phenotype for each frame.

We previously showed that She1 regulates dynein activity (Markus et al., 2012b), but the domain required for this function is unknown. We generated s*he1* alleles expressing N- and C-terminal truncations of the protein at the chromosomal locus (Fig. 1 A) and assayed for dynein-regulating function using GFP-Tub1–expressing cells. Time-lapse videos were captured, and cells were scored for showing enhanced spindle movements (Fig. 1 B) or increased astral MT detachment from the spindle pole body (Fig. S2 A), both of which report on *she1Δ* phenotypes resulting from hyperactivity of cortical dynein (Bergman et al., 2012; Markus et al., 2012b). She1 contains two neighboring N-terminal motifs, hereafter termed PISH1 and PISH2 (for Present in She1 1 and 2), which were identified by BLAST (Basic Local Alignment Search Tool) searches and sequence alignments with its fungal homologues (Fig. S1). We found that *she1-ΔN89* and *she1-ΔN126*, the alleles lacking PISH1 and both PISH1 and 2, respectively, displayed normal levels of dynein regulation compared with WT *SHE1*. Further truncation from the N terminus (*she1-ΔN212*), along with truncations from the C terminus (*she1-N212*, *she1-N126*, and *she1-N89*), resulted in hyperactivity of cortical dynein, indicating that the C-terminal domain encompassing 127–338 aa was required for dynein-regulating activity (Fig. 1 B). Immunoblotting of she1-N212 and she1-ΔN89 showed that the difference in their activity could not be attributed to altered expression levels or protein stability (Fig. S2 B).

### Establishing a new role for She1 in the maintenance of metaphase spindle stability

She1 localizes to the spindle throughout mitosis (Fig. S2 C; Woodruff et al., 2009), but whether it has any role on the metaphase spindle remains unknown (Woodruff et al., 2010). We developed a live-cell assay to monitor spindle behavior in Cdc20-depleted metaphase cells. Whereas WT spindles appeared rigid and moved between mother and bud without any changes in shape and length (Video 1, left; and Fig. 1 C), a high percentage of *she1Δ* spindles (32.1%) displayed a bent or collapsed spindle phenotype (Video 1, middle; and Fig. 1 C). Because the collapsed phenotype was often observed when a bent spindle buckled (11 out of 26; Video 3, right), the frequencies of observing spindle bending and collapse events were combined (Fig. 1 C, right). Additionally, a small but significant percentage of *she1Δ* spindles (11.1%) showed a broken spindle phenotype that appeared to be different from the bent/collapsed phenotype. Broken spindles were observed as two separate GFP-Tub1 spindle halves (with a clear gap) that moved independently (Video 1, right) or stretched apart when they were pulled through the bud neck (e.g., time = 9–10 min for *she1Δ* in Fig. 1 C), possibly by hyperactive cortical dynein. In agreement with this notion, the broken spindle phenotype in *she1Δ* cells was reduced by 3.6-fold upon deletion of *DYN1* (12.0 to 3.3%; Fig. S2 D). Moreover, addition of the *kar9Δ* mutation, which causes dramatic back and forth spindle movements across the bud neck (Yeh et al., 2000; Moore et al., 2009a), enhanced the broken spindle phenotype seen in *she1Δ* cells (12.0 to 23.3%; Fig. S2 D), suggesting that She1 helps maintain spindle integrity during dynein-mediated spindle movements.

We found that *she1-ΔN89* and *she1-ΔN126* alleles, which displayed dynein-regulating activity (Fig. 1 B), rescued the broken spindle phenotype observed in *she1Δ* (from 11.1% to 0% and 3.6% for *she1Δ*, *she1-ΔN89*, and *she1-ΔN126*, respectively; Fig. 1 C). However, cells expressing these alleles still displayed a high level of the bent/collapsed spindle phenotype like *she1Δ* (32.1% vs. 35.7% and 32.9% for *she1Δ*, *she1-ΔN89*, and *she1-ΔN126*, respectively; Fig. 1 C and Video 2), indicating a specific spindle defect independent of dynein. We found that the spindle defects in *she1Δ* could not be attributed to the increase in spindle MTs upon Cdc20 depletion (Fig. S2 E; O’Toole et al., 1997). Furthermore, we confirmed that the bent/collapsed spindle phenotype was not caused by the metaphase arrest, as imaging of cycling cells also revealed spindle bending before anaphase (Fig. S2 F). In contrast, *she1-N212* cells, which exhibited hyperactivity of cortical dynein (Fig. 1 B), showed a similar frequency of the broken spindle phenotype as *she1Δ* (13.3 vs. 11.1%; Fig. 1 C and Video 2) but a significant decrease in the bent/collapsed spindle phenotype (from 32.1 to 10%; Fig. 1 C). Thus, our data revealed that the spindle stabilization function of She1 requires (a) the C-terminal dynein-regulating domain, deletion of which caused a broken spindle phenotype attributed to an enhanced cortical dynein activity, and (b) the N-terminal PISH1/2 motifs, deletion of which caused a bent/collapsed spindle phenotype resulting from an uncharacterized spindle defect that appeared to be independent of cortical dynein.

Based on colocalization with the kinetochore marker Nuf2-mCherry (Fig. 1 D and Video 3, middle) and the spindle cross-linker Ase1-3GFP (Fig. S2 H), we identified the arched MTs in the bent spindles as ipMTs. Colocalization with the nuclear envelope marker Nup133-TagRFP confirmed that the bent and collapsed MTs were encompassed within the nucleus (Video 4 and Fig. S2 H). During metaphase block, ipMT buckling could cause a bent spindle to collapse (Video 3, right). Intriguingly, collapsed spindles could re-establish bipolarity and revert into normal-looking spindles (Video 5 and Fig. 1 E; *she1Δ*, 6 of 11; *she1-ΔN89*, 5 of 9; *she1-ΔN126*, 4 of 10), indicating that the abnormal spindle phenotypes could be caused by an impaired ipMT stability.

**Figure 2.**
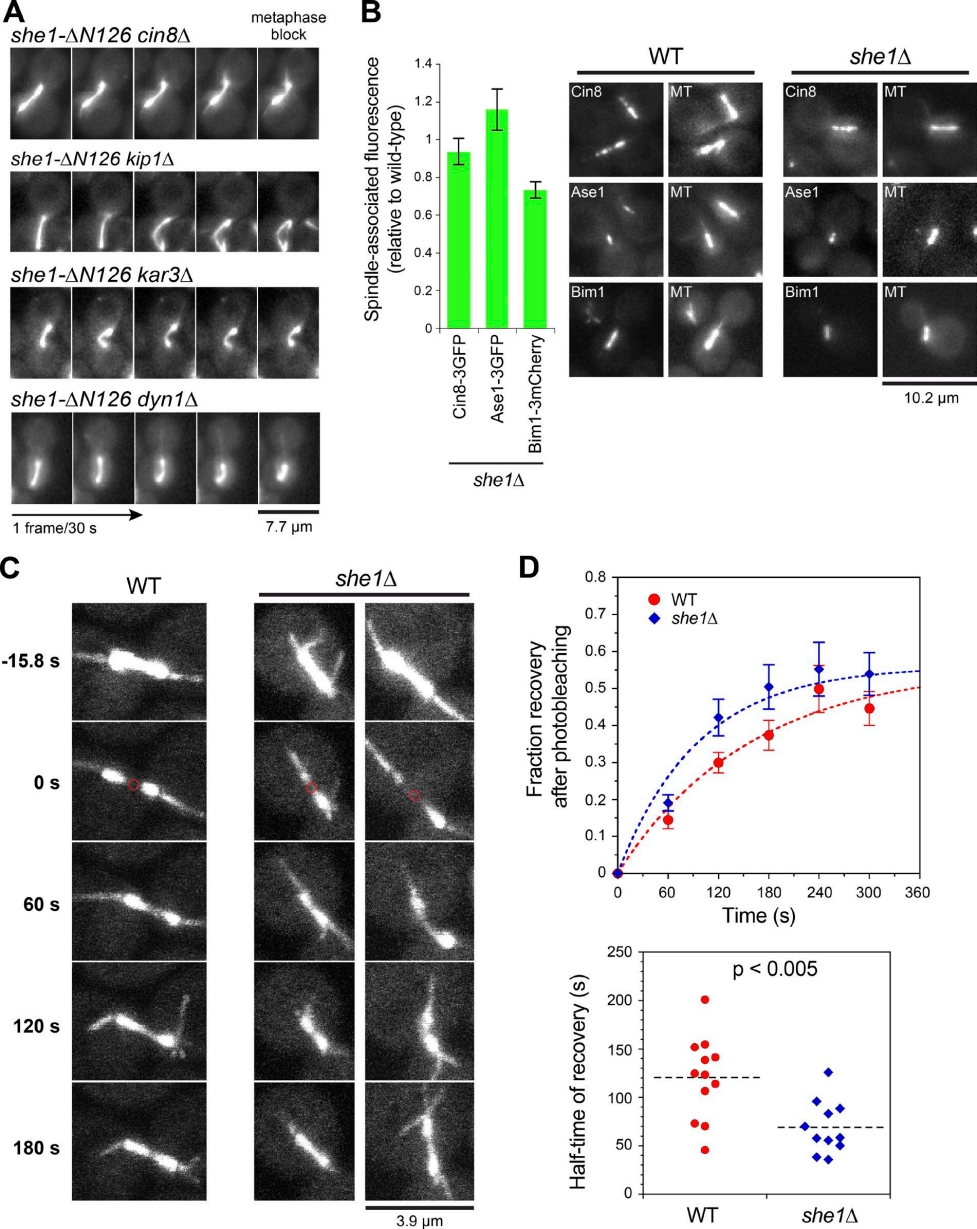
**She1 deletion reduces spindle-associated Bim1 and increases spindle MT dynamics.** (A) Video frames showing bent spindle phenotype in *MET3-CDC20 GFP-TUB1 she1-ΔN126* cells with *cin8Δ*, *kip1Δ*, *kar3Δ*, or *dyn1Δ* (*n* ≥ 50 cells). (B) Quantification and representative images of spindle-associated Cin8-3GFP, Ase1-3GFP, and Bim1-3mCherry in Cdc20-depleted *she1Δ* cells relative to WT (*n* ≥ 45 spindles). (C) Representative FRAP images of GFP-Tub1 spindles in WT and *she1Δ* cells arrested in metaphase. Photobleached areas are indicated by circles at 0 s. (D, top) Mean fractional recovery after photobleaching for 12 WT and 11 *she1Δ* spindles. Error bars are SEM. (Bottom) Quantification of half-maximal recovery time. P < 0.005 by unpaired *t* test.

Bent and collapsed spindles could occur if kinesin-5 motors prematurely slid ipMTs apart in the *she1-ΔN126* metaphase cells (Khmelinskii et al., 2009). If so, reducing the activity of Cin8 and Kip1 should suppress the *she1-ΔN126* spindle phenotype. However, we observed no change in the frequency of bent metaphase spindles upon deletion of *CIN8* or *KIP1* (P > 0.7; Fig. 2 A and Fig. S3 A). We next tested whether loss of Kar3 could lead to the bent spindle morphology. A recent study showed that Kar3 contributes to MT alignment in the metaphase spindle (Hepperla et al., 2014). Unlike *she1-ΔN126* spindles, *kar3Δ* spindles did not exhibit a similar bent phenotype (Fig. S3 B). Moreover, we observed no change in the frequency of bent spindles in *she1Δ kar3Δ*, *she1-ΔN89 kar3Δ*, and *she1-ΔN126 kar3Δ* double mutants compared with *she1Δ*, *she1-ΔN89*, and *she1-ΔN126* single mutants (Fig. S3 B vs. Fig. 1 C). These results suggest that the bent spindle morphology is not due to loss of MT alignment by Kar3. Additionally, deletion of *DYN1* did not abolish the *she1-ΔN126* spindle phenotype (P = 0.35; Fig. 2 A and Fig. S3 A), confirming that the observed bending was independent of cortical pulling forces generated by hyperactive dynein.

### *SHE1* deletion reduced spindle-associated Bim1 and increased spindle MT dynamics contributing to chromosome segregation errors

Bent and collapsed phenotypes could occur if there are cross-linking defects. We examined the localization of spindle cross-linkers and stabilizers Ase1, Cin8, and Bim1 (Kotwaliwale et al., 2007; Gardner et al., 2008; Khmelinskii et al., 2009; Zimniak et al., 2009) to the *she1Δ* metaphase spindle relative to the WT metaphase spindle. The fluorescence intensity of Bim1-3mCherry was slightly reduced (by 26.9%; P < 0.0002; Fig. 2 B), whereas the levels of Ase1-3GFP and Cin8-3GFP did not change significantly (P > 0.3; Fig. 2 B). The observed reduction for Bim1-3mCherry might explain why *she1Δ* spindles were less stable, given that Bim1 was known to promote ipMT growth (Gardner et al., 2008).

To understand how spindle MT dynamics were affected in the absence of She1, we performed FRAP analysis on metaphase spindles marked with GFP-Tub1. Different regions of the spindle were selectively targeted for photobleaching—either one half of the spindle or the middle of the spindle—to assess the dynamics of different sets of MTs. In half-spindle bleaching experiments, we did not detect a significant difference in the recovery kinetics between *she1Δ* and WT spindles (*t*_1/2_ = 62 ± 4 s vs. 59 ± 6 s; P > 0.7; Fig. S3 C). However, in midspindle bleaching experiments, we observed a 1.7× faster recovery for *she1Δ* spindles compared with WT spindles (*t*_1/2_ = 69 ± 8 s vs. 120 ± 12 s; P < 0.005; Fig. 2, C and D). Similar results were obtained for midspindle FRAP of *she1-ΔN126* metaphase spindles (*t*_1/2_ = 46 ± 4 s; P < 0.0003; Fig. S3 D), indicating that the increase in tubulin turnover could not be attributed to hyperactivity of cortical dynein. Because the midspindle was mostly composed of ipMTs, our data suggested that *SHE1* deletion caused a specific increase in ipMT dynamics.

Given the observed spindle defects, we wondered whether loss of She1 could lead to elevated chromosome segregation errors. We tested this possibility by arresting haploid yeast in G1 and visualizing fluorescently marked chromosome IV (Chr IV). We noticed that *she1Δ* cells often possessed two Chr IVs (two GFP dots) compared with WT cells, which is consistent with a previous study (Woodruff et al., 2009). Depletion of dynein in *she1Δ* cells dramatically reduced the unequal chromosome distribution phenotype seen in G1 cells (Fig. S2 G; *she1Δ*, 18.4%; *she1Δ dyn1Δ*, 3.1%). These data support the notion that She1 helps maintain spindle integrity during dynein-mediated spindle movement to ensure accurate chromosome segregation between the mother and daughter cell.

### She1 cross-linked MTs via its C-terminal 194–338 domain

**Figure 3.**
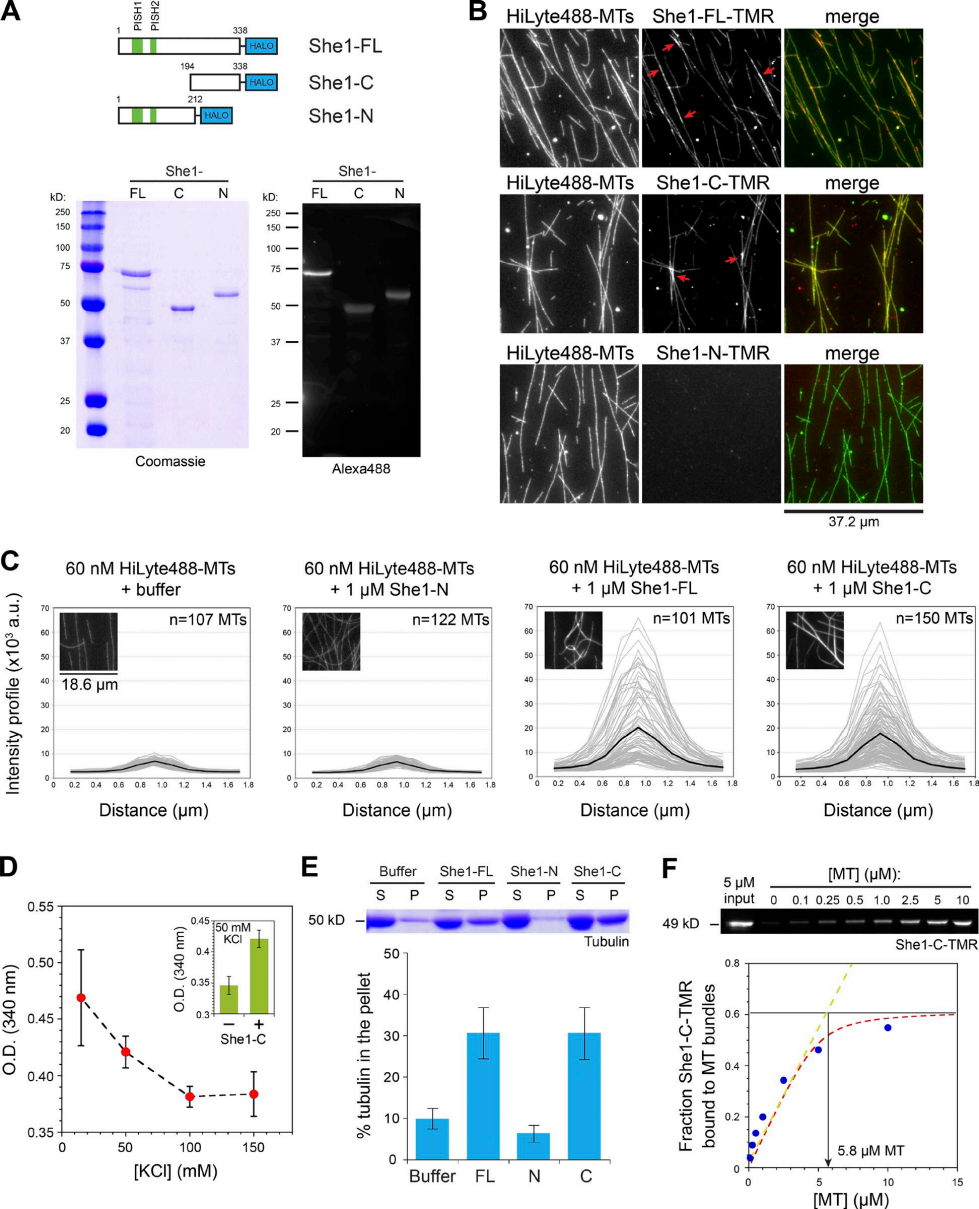
**She1 binds and promotes MT cross-linking via its C-terminal domain.** (A) Schematic diagram and gel images of recombinant She1 constructs. (B) Wide-field images of TMR-labeled She1 constructs incubated with HiLyte 488–labeled, Taxol-stabilized MTs. Red arrows indicate regions of cross-linked MTs with high signals of She1-FL–TMR or She1-C–TMR. (C) Intensity profiles of individual HiLyte 488–labeled MT structures (gray lines) in the presence of She1-FL, She1-C, She1-N, or buffer alone. Mean profiles are shown as a black line. Insets show representative images used for quantification. (D) Turbidity decreases as the ionic strength is increased for a solution containing 10 µM MTs and 8 µM She1-C. Inset shows absorbance of 10 µM MTs with and without added 8 µM She1-C at 50 mM KCl. (E) Low-speed pelleting of 5 µM Taxol-stabilized MTs (at 2,348 *g*) with and without 1 µM She1-FL, She1-N, or She1-C. S, supernatant; P, pellet. (D and E) Error bars are SEM for three experiments. (F) Low-speed copelleting of 5 µM She1-C–TMR with varying concentrations of Taxol-stabilized MTs (at 15,871 *g*). The fraction of bound She1-C–TMR was quantified by SDS gel (top) and fitted with a quadratic equation as a function of MT concentration (red dotted curve). Linear regression of the initial slope is indicated by a green dotted line.

Previous studies showed that She1 can bind MTs directly (Bergman et al., 2012; Markus et al., 2012b), but the domain required for this activity is unknown. To detect new activity and to understand how She1 stabilized spindle MTs, we biochemically isolated recombinant HALO constructs of She1 from bacteria and characterized their interactions with MTs. Fig. 3 A shows purification of full-length She1 (She1-FL) and two soluble constructs containing the N-terminal 1–212 aa or the C-terminal 194–338 aa of She1 (She1-N and She1-C, respectively). To assay for MT binding, tetramethylrhodamine (TMR)-labeled She1 constructs were mixed with Taxol-stabilized MTs, and their interaction was assessed by wide-field microscopy. Fig. 3 B shows that She1-FL and She1-C, but not She1-N, were bound to the MTs (mean TMR fluorescence on MTs = 2.2 ± 0.3 × 10^4^ a.u. and 2.1 ± 0.5 × 10^4^ a.u. vs. 104 ± 218 a.u., respectively), indicating that the MT-binding site of She1 is located within the C-terminal 194–338 aa.

Interestingly, while imaging MTs pre-incubated with She1-FL or She1-C, we detected an increased level of cross-linked or bundled MTs and an apparent accumulation of She1-FL and She1-C at regions of MT overlap (Fig. 3 B, red arrows), suggesting that She1 might possess an MT cross-linking or bundling activity. To address this quantitatively, we compared the MT cross-sectional intensity by measuring the fluorescence profile across individual MT structures observed in the microscopy-based binding assay. Fig. 3 C shows that HiLyte 488–labeled MT structures observed in the presence of She1-FL or She1-C have, on average, a significantly higher intensity profile compared with the MTs in the presence of She1-N or buffer control (mean peak intensities = 20,114 ± 15,053 a.u. for She1-FL; 17,779 ± 10,812 a.u. for She1-C; 6,661 ± 1,241 a.u. for She1-N; and 6,968 ± 1,023 a.u. for buffer alone). These data indicated that She1-FL and She1-C possess an MT cross-linking activity.

Next, we confirmed that She1-C could cross-link MTs using bulk assays. We found that She1-C increased the turbidity of Taxol-stabilized MTs (Fig. 3 D, inset), as determined by absorbance at 340 nm. The increase in turbidity was interpreted as an increase in light scattering through cross-linking of MTs (Stumpff et al., 2007; Mirigian et al., 2013). This effect decreased as the ionic strength was increased (Fig. 3 D), indicating that the cross-linking activity was salt sensitive. Additionally, using a microfuge-based pelleting assay, we found that the amount of tubulin that pelleted in the presence of She1-FL or She1-C was significantly higher when compared with She1-N or the buffer control (Fig. 3 E). Because the MTs were sedimented at low speed, the pellet fraction represented MTs that were cross-linked by She1-FL or She1-C. Furthermore, titration of TMR-labeled She1-C with increasing concentrations of MTs revealed an ~1:1 ratio of bound She1-C–TMR to tubulin at the saturation point (Fig. 3 F), indicating that She1 might self-associate to cross-link MTs.

### She1 formed ring-shaped or globular complexes and elongated structures

**Figure 4.**
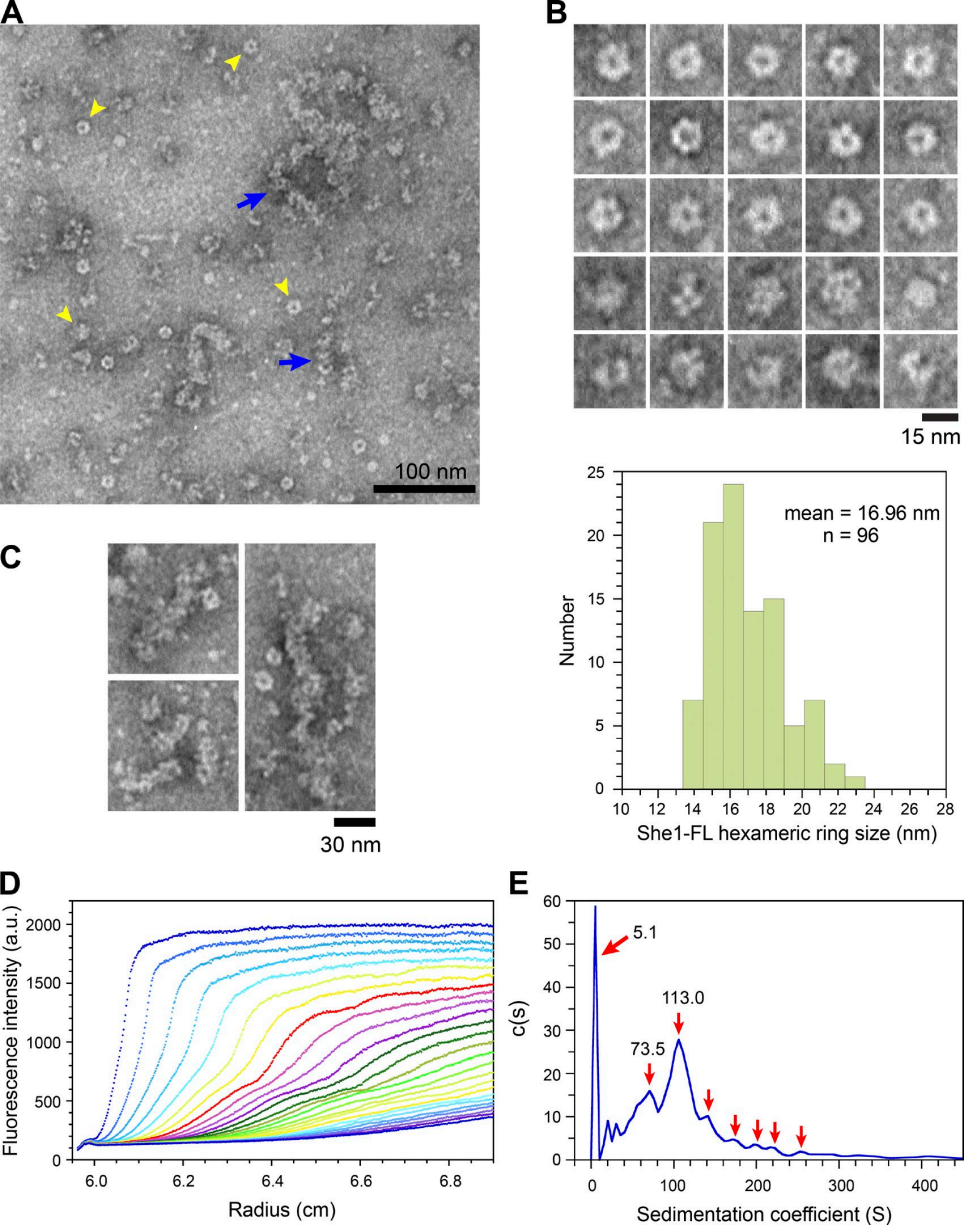
**EM and analytical ultracentrifugation reveal oligomerization of She1.** (A–C) Purified She1-FL at 4 µM was visualized by EM using negative stain. (A) Field showing individual rings (yellow arrowheads) and longitudinal assemblies of rings (blue arrows). (B) Gallery of individual She1-FL ring and globular complexes. (Bottom) Histogram showing distribution of the caliper length of individual She-FL particles. (C) Gallery of elongated chain-like structures assembled by longitudinal association of ring-shaped or globular complexes. (D) Velocity sedimentation of purified 0.5 µM Alexa Fluor 488–labeled She1-FL at 20,000 rpm detected by fluorescence optical analytical ultracentrifugation. Radial scans showing multiple sedimenting boundaries indicating the existence of multiple species. (E) Sedimentation coefficient distribution, *c*(s), for the velocity experiment shown in D.

We subsequently tested for self-association of She1 by EM and analytical ultracentrifugation. When visualized by EM using negative stain, purified She1-FL showed prominent ring-shaped and globular particles (Fig. 4 A, arrowheads) as well as elongated structures that appeared to be made up of chains of ring-shaped or globular subunits (Fig. 4 A, arrows). Fig. 4 B (top) shows representative morphologies of the ring-shaped and globular particles. Approximately half of the particles (52.1%, *n* = 73) displayed a distinct six-lobed, hexameric morphology with a clearly visible central cavity. Nearly one third (28.8%) were globular in appearance, whereas the remaining 19.1% had a splayed, open shape with a visible central cleft. The mean diameter of the six-lobed hexameric ring particles was 17 ± 2 nm (Fig. 4 B, bottom; *n* = 96 molecules from two separate preparations). The particles could be readily seen in the vicinity of elongated chain-like structures (Fig. 4 C), suggesting that ring complexes could associate longitudinally to form higher-order structures of variable length. Although it is unclear whether these structures can bind and cross-link MTs at this point, the aforementioned observations demonstrate that She1 can self-associate into oligomers and that this behavior can explain why we observed MT cross-linking activity in the biochemical assays (Fig. 3, C–F).

Consistent with the EM data, sedimentation velocity experiments revealed polydispersity in purified samples of She1-FL. Fig. 4 D shows sedimentation profiles of Alexa Fluor 488–labeled She1-FL with multiple sedimenting boundaries indicating the existence of multiple species. The sedimentation coefficient (S) distribution plot (Fig. 4 E) indicated major peaks at 5.1, 73.5, and 113.0 S, as well as multiple smaller peaks at higher S values. As a control, purified Alexa Fluor 488–labeled She1-N sedimented as a single homogeneous peak at 3.91 S (Fig. S3, E and F), indicating that self-association of She1-FL could not be attributed to the HALO tag. Furthermore, analytical gel filtration analysis of yeast extract expressing 13myc-tagged She1 (not depicted) indicated that the protein migrated as a broad peak with an apparent molecular mass (>440 kD) markedly larger than the molecular mass calculated from its amino acid composition (58.4 kD), suggesting that the native protein might also be assembled into oligomers or higher-order structures, as observed for the recombinant protein by EM.

### Loss of She1 caused premature Ipl1 localization to the metaphase spindle

**Figure 5.**
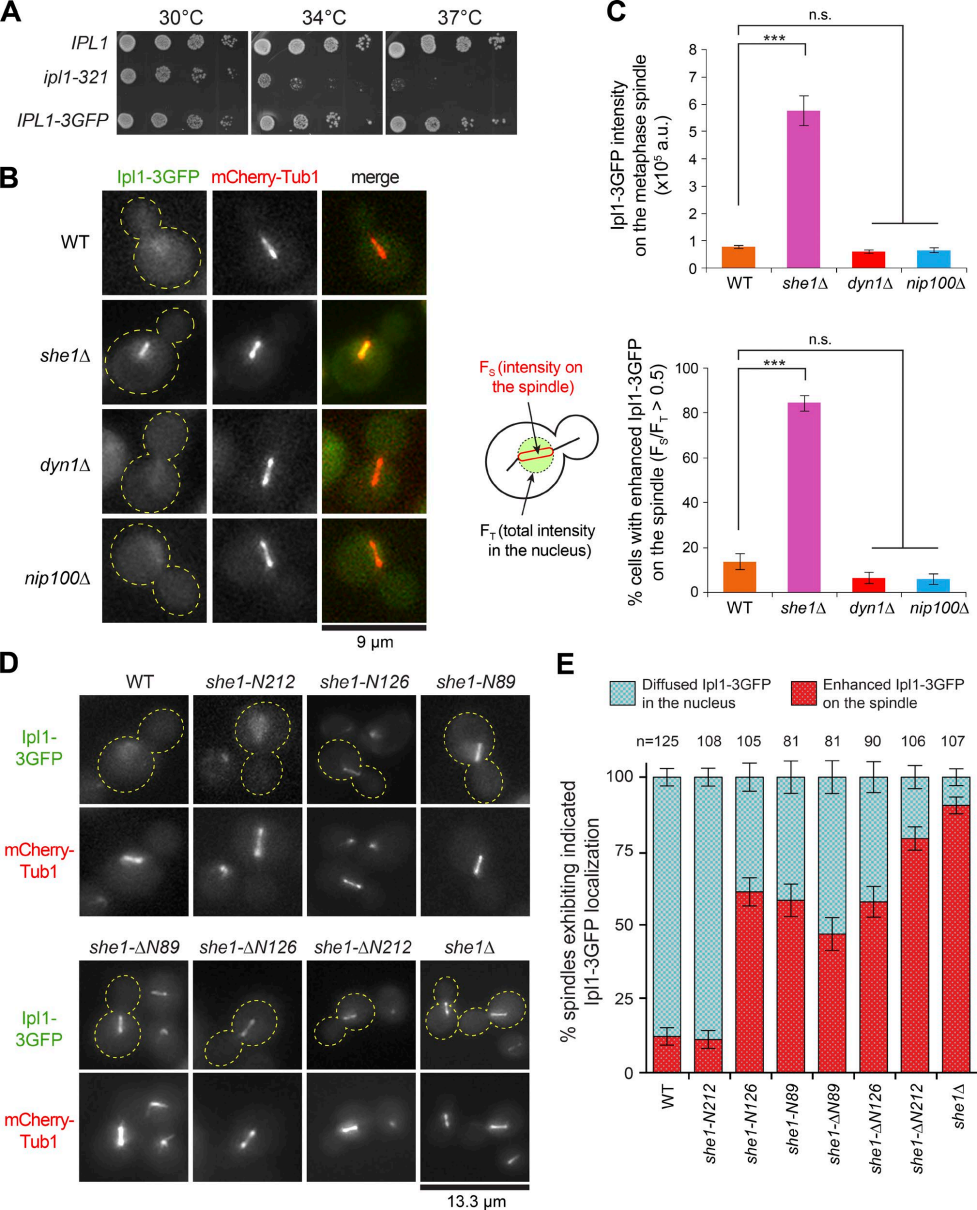
**Ipl1/Aurora B is prematurely targeted to the metaphase spindle in *she1Δ*.** (A) Spotting growth assays on rich media showing *IPL1-3GFP* is functional. (B) Ipl1-3GFP and mCherry-Tub1 in metaphase-arrested WT, *she1Δ*, *dyn1Δ*, and *nip100Δ* cells. (C) Quantification of spindle-associated Ipl1-3GFP intensity and percentage of cells with an enhanced Ipl1-3GFP phenotype during metaphase block. Error bars are SEM (top) and standard error of proportion (bottom). ***, P < 0.0001; n.s., P ≥ 0.01 by unpaired *t* test (*n* ≥ 94 for each). (D) Localization of Ipl1-3GFP in cells expressing the indicated *she1* alleles. (E) Quantification of cells exhibiting diffused Ipl1-3GFP in the nucleus or enhanced Ipl1-3GFP on the spindle. Error bars are standard error of proportion.

Our biochemical and biophysical experiments suggest that She1 functions as an MT cross-linker on the metaphase spindle. Although the C-terminal domain of She1 harbors the MT cross-linking activity (Fig. 3, C–F), this domain alone did not appear to be sufficient for rescuing the spindle stability defects observed in *she1Δ* (Fig. 1 C, *she1-ΔN126* vs. *she1Δ*), suggesting that its activity might be regulated in vivo. We considered the possibility that Ipl1, the yeast Aurora B kinase, might regulate the MT cross-linking activity of She1. This hypothesis is supported by previous work showing that Ipl1 phosphorylation regulates She1 association with MTs in vitro (Markus et al., 2012b). Our results in Fig. 2 B showing a decrease in spindle-associated Bim1 in *she1Δ* cells further suggested that Ipl1 activity might be altered on the *she1Δ* spindles because Ipl1 phosphorylation of Bim1 was known to reduce its spindle association (Zimniak et al., 2009; Woodruff et al., 2010). To investigate this, we first asked whether loss of She1 affected Ipl1 targeting to the spindle. We tagged the endogenous copy of Ipl1 with 3GFP at the C terminus. The tagged protein was functional (Fig. 5 A). In WT cells, Ipl1-3GFP displayed a diffuse nuclear signal during metaphase (Fig. 5 B) and localized to the spindle only upon anaphase onset (Fig. S4, A and B; and Video 6) as previously reported (Tanaka et al., 2002; Zimniak et al., 2012). Remarkably, in *she1Δ* cells, Ipl1-3GFP exhibited a clear spindle association during metaphase, with visibly reduced or undetectable nuclear background signal (Fig. 5 B). Its localization to *she1Δ* spindles during anaphase appeared to be unaffected (Fig. S4 C), suggesting that Ipl1 was prematurely loaded to the metaphase spindle in the absence of She1. Intensity measurements showed that the level of Ipl1-3GFP on the Cdc20-depleted metaphase spindles was enhanced by 7.5-fold in the *she1Δ* background relative to the WT background (Fig. 5 C, top). This increase was reflected by a high percentage of *she1Δ* metaphase-blocked cells exhibiting an enhanced Ipl1-3GFP phenotype (defined as showing >50% of Ipl1-3GFP signal within the nucleus loaded on the spindle): 84.3% of *she1Δ* versus 13.7% of WT cells (Fig. 5 C, bottom). The same Ipl1-3GFP enhancement was also observed when *she1Δ* cells were arrested at preanaphase by hydroxyurea (not depicted). These results demonstrate that loss of She1 caused Ipl1 to associate prematurely with the metaphase spindle.

We wondered whether the observed premature spindle localization of Ipl1-3GFP in *she1Δ* was caused by the disruption of dynein pathways. We tested two dynein pathway components, Dyn1 (dynein heavy chain) and Nip100 (dynactin p150^Glued^ subunit). Like WT cells, *nip100Δ* and *dyn1Δ* mutants displayed a diffuse nuclear signal of Ipl1-3GFP (Fig. 5 B), with no enhanced Ipl1-3GFP phenotype on the metaphase spindle (Fig. 5 C), indicating that the observed premature spindle localization of Ipl1-3GFP in the *she1Δ* mutant was specific to loss of She1 function on the spindle.

We next identified the She1 domain required for regulating Ipl1 localization. Whereas *she1-ΔN89*, *she1-ΔN126*, and *she1-ΔN212* mutants exhibited an enhanced Ipl1-3GFP phenotype like *she1Δ* cells (Fig. 5, D and E), the *she1-N212* mutant displayed a diffuse nuclear Ipl1-3GFP localization like WT cells (Fig. 5, D and E), indicating that the N-terminal 1–212 aa containing the PISH1 and 2 motifs were required for regulating Ipl1 association with the spindle. Furthermore, mRuby2 tagging of she1-N212 revealed that spindle localization was not required for preventing premature loading of Ipl1 (Fig. S3 G). Because relocalization of Ipl1 to the metaphase spindle (as a component of the chromosomal passenger complex [CPC]) was previously observed in cells exhibiting reduced phosphorylation of Sli15 (the yeast INCENP; Pereira and Schiebel, 2003; Nakajima et al., 2011), and because Sli15 interacted with She1 in two-hybrid assays (Wong et al., 2007), we wondered whether the enhanced Ipl1-3GFP phenotype in *she1-ΔN126* was caused by an altered Sli15–She1 interaction. To investigate this possibility, we biochemically isolated recombinant GST-Sli15 from bacteria and used it to pull down 13myc-tagged She1 and she1-ΔN126 from yeast cell lysates. GST-Sli15 pulled down a significantly reduced amount of she1-ΔN126-13myc compared with full-length She1-13myc (P < 0.05; Fig. S4 D), suggesting that the N-terminal PISH1 and 2 motifs were required for normal interaction with Sli15. When combined with our domain analysis for the Ipl1 loading phenotype (Fig. 5, D and E), this result suggests that She1 regulates Ipl1/CPC recruitment through Sli15 (see Discussion). Collectively, our data show that uncontrolled loading of Ipl1 onto the metaphase spindle might contribute to the *she1-ΔN126* spindle phenotype observed in Fig. 1 C.

### Ipl1 phosphorylation inhibited She1 spindle localization and MT cross-linking activity

**Figure 6.**
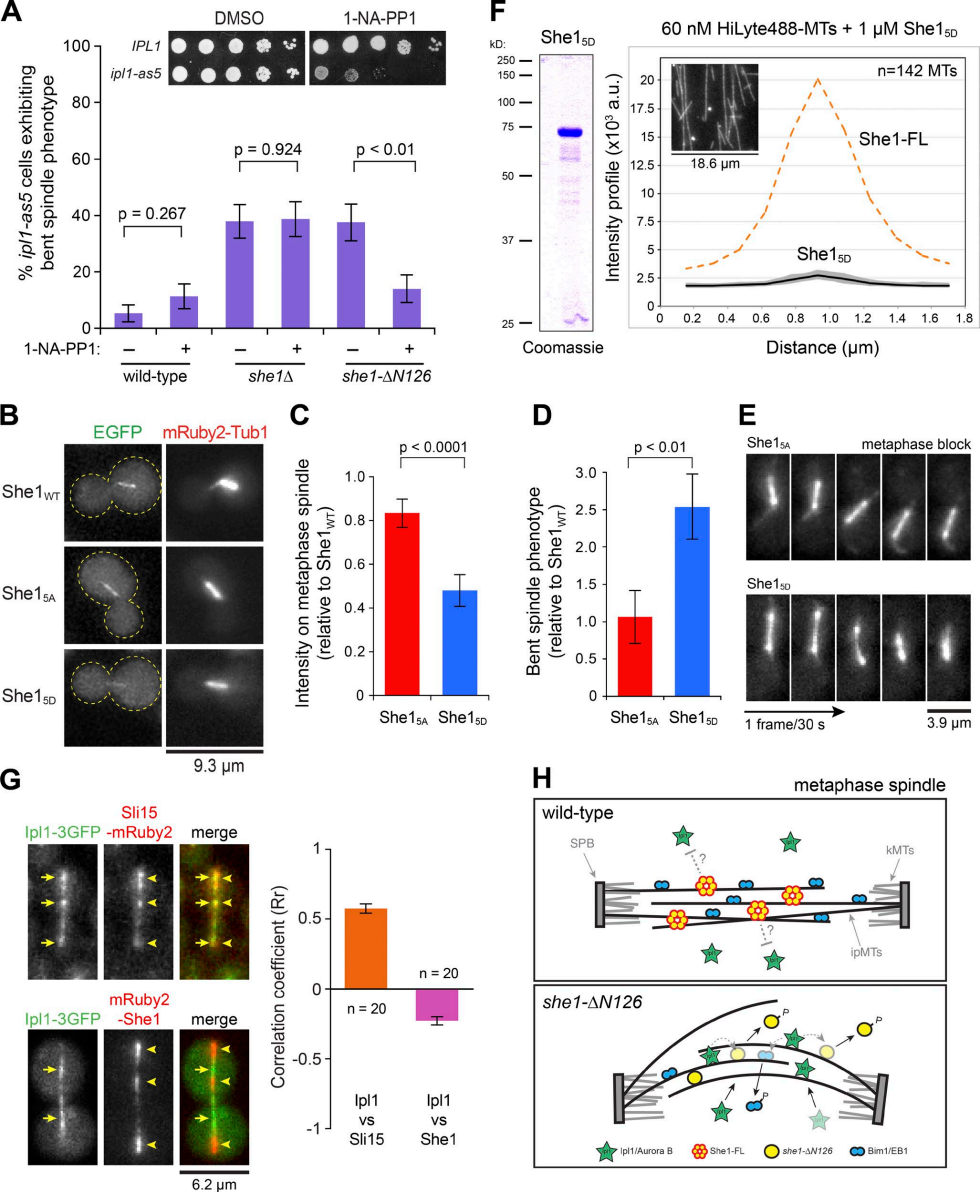
**Ipl1 phosphorylation negatively regulates She1 spindle localization and MT cross-linking activity.** (A) Percentage of metaphase-arrested spindles exhibiting the bent phenotype in WT, *she1Δ*, and *she1-ΔN126* cells treated with DMSO (−) or 50 µM 1-NA-PP1 (+). Error bars are standard error of proportion (*n* ≥ 50 for each). Inset, sensitivity of *ipl1-as5* to 1-NA-PP1. (B) Localization of EGFP-tagged She1_WT_, She1_5A_, and She1_5D_ in Cdc20-depleted mRuby2-Tub1 cells. (C) Quantification of spindle-associated EGFP-She1_5A_ and She1_5D_ relative to She1_WT_. Error bars are SEM (*n* ≥ 70 spindles for each). P < 0.0001 by unpaired *t* test. (D) Quantification of the bent spindle phenotype in She1_5A_ and She1_5D_ relative to She1_WT_. Error bars are standard error of proportion (*n* ≥ 80 cells each, P < 0.01 by *t* test). (E) Representative video frames of metaphase spindles for quantification in D. (F) She1_5D_ purified from *Escherichia coli*. (Right) Intensity profiles for 142 cross sections of MTs (gray lines). Inset, representative image. Black line = mean intensity. Mean intensity for She1-FL (orange dashed line) from Fig. 3 C is overlaid for comparison. (G) Colocalization correlation analysis of Ipl1-3GFP with Sli15-mRuby2 or mRuby2-She1 along a WT anaphase spindle. Yellow arrows indicate Ipl1-3GFP puncta, whereas arrowheads indicate Sli15-mRuby2 (top) or mRuby2-She1 (bottom) puncta. Error bars are SEM. (H) Model showing She1 function on the metaphase spindle. We propose that She1 cross-links ipMTs and prevents premature loading of Ipl1 (see Discussion). In *she1-ΔN126* cells, Ipl1 prematurely loads onto the metaphase spindle, causing a decrease in the levels of both She1 and Bim1, resulting in the bent spindle phenotype and increased ipMT dynamics. kMTs, kinetochore MTs. SPB, spindle pole body.

We next tested whether premature loading of Ipl1 onto the metaphase spindle could phosphorylate and inhibit the MT cross-linking activity of She1. To investigate this, we asked whether inactivation of Ipl1 would rescue the *she1-ΔN126* bent spindle phenotype. We monitored metaphase spindle behavior in cells carrying an analogue-sensitive *ipl1* allele (*ipl1-as5*). Growth assays confirmed that *ipl1-as5* was inhibited by the ATP analogue 1-naphthyl-pyrazolo[3,4-d]pyrimidine (1-NA-PP1; Fig. 6 A, inset; Pinsky et al., 2006). We found that the frequency of observing bent metaphase spindles in *she1-ΔN126 ipl1-as5* cells was significantly reduced by the 1-NA-PP1 inhibitor (P < 0.01) compared with the bent spindle frequency observed in DMSO-treated cells (Fig. 6 A) or untreated *she1-ΔN126* cells carrying WT *IPL1* (Fig. 1 C). In control *she1Δ ipl1-as5* cells, 1-NA-PP1 treatment did not rescue the bent spindle phenotype (Fig. 6 A), which was expected given that She1 was absent in these cells. Furthermore, overexpression of *she1-ΔN126* or *she1-ΔN89* (using a *GAL1* promoter [*GAL1*p]) significantly decreased the bent spindle phenotype (35.1 to 15.8% and 26.9 to 9.4%, respectively; Fig. S4 E), supporting the notion that the truncated She1 protein was a downstream substrate of the prematurely loaded Ipl1 kinase. These findings showed that Ipl1, if prematurely loaded to the metaphase spindle, could negatively regulate spindle stabilization by phosphorylating She1.

We next asked what the consequences would be of Ipl1 phosphorylation on She1 spindle localization and metaphase spindle stabilization function in an otherwise WT cell (i.e., without premature loading of Ipl1). We made use of the established *she1_5A_* and *she1_5D_* mutants, in which all five putative Ipl1 consensus sites had been mutated to alanine (to prevent phosphorylation) or aspartate (to mimic constitutive phosphorylation; Woodruff et al., 2010; Markus et al., 2012b). We altered the genomic locus to express an N-terminal EGFP fusion of She1_WT_, She1_5A_, or She1_5D_ under the control of its endogenous promoter (Fig. 6 B). The tagged EGFP-She1_WT_ protein was functional, rescuing spindle phenotypes and dynein hyperactivity in *she1Δ* (not depicted). We found that the fluorescence intensity of EGFP-She1_5A_ on Cdc20-depleted metaphase spindles was unaffected compared with EGFP-She1_WT_, but the fluorescence intensity for EGFP-She1_5D_ was reduced significantly (by ~2.1-fold; Fig. 6 C), indicating a negative regulation on spindle localization by Ipl1-mediated phosphorylation. Furthermore, the reduction in EGFP-She1_5D_ spindle localization was accompanied by an ~2.5-fold increase in the frequency of the observed bent spindle phenotype (P < 0.01; Fig. 6, D and E), whereas EGFP-She1_5A_ spindles showed no spindle defects relative to WT spindles (P = 0.90; Fig. 6, D and E). These experiments indicated that Ipl1-mediated phosphorylation could negatively regulate She1 by reducing its localization and function on the metaphase spindle.

To further examine the effects of Ipl1 phosphorylation on MT cross-linking activity in vitro, we expressed and purified recombinant She1_5D_ from bacteria (Fig. 6 F, left) and assessed its ability to cross-link Taxol-stabilized, HiLyte 488–labeled MTs by microscopy. We found that She1_5D_ exhibited no MT cross-linking activity, as indicated by a low-intensity profile of MT cross sections compared with WT She1-FL (Fig. 6 F, right; mean peak intensity of 2,746 ± 167 a.u. vs. 20,114 ± 15,053 a.u.; P < 0.0001), further indicating that the negative regulation by Ipl1 could be mediated through inhibition of the MT cross-linking activity of She1.

Our experiments showed that She1 spindle localization was reduced by Ipl1 phosphorylation (Fig. 6, B and C), whereas Ipl1 relocalized to the spindle only upon anaphase onset (Fig. S4, A and B), suggesting that Ipl1 and She1 localization on the spindle might be anticorrelated in anaphase. Interestingly, video analysis of Ipl1-3GFP and mRuby2-She1 on the anaphase spindles supported this notion (Video 7). After anaphase onset, both Ipl1-3GFP and mRuby2-She1 displayed a discontinuous pattern but nonoverlapping localization along the elongating anaphase spindle. Additionally, quantitative correlation analysis revealed a negative Pearson’s coefficient (Rr = −0.23 ± 0.03; *n* = 20 spindles) between Ipl1-3GFP and mRuby2-She1 on the anaphase spindle (Fig. 6 G), indicating a clear anticolocalization. In comparison, correlation analysis of Ipl1-3GFP with Sli15-mRuby2, which associated with Ipl1 as part of the CPC (Carmena et al., 2012), yielded a positive Pearson’s correlation coefficient (Rr = 0.51 ± 0.03; *n* = 20 spindles) for colocalization of the two proteins on the anaphase spindle. Thus, when combined with our in vitro data, the aforementioned observations suggest that Ipl1 loading onto the spindle during the metaphase–anaphase transition might serve to inactivate MT cross-linking and to dissociate She1 from the spindle. Our results indicate that She1’s role on the metaphase spindle is regulated by spatial and temporal loading of Ipl1 kinase during anaphase.

## Discussion

In conclusion, we propose that the mitotic spindle protein She1 functions as an MT cross-linking protein to maintain proper spindle stability during metaphase (Fig. 6 H). There are three major lines of evidence supporting this conclusion: (1) *she1-ΔN89* and *she1-ΔN126* cells display dynein-independent spindle defects, (2) She1 cross-links MTs in vitro, and (3) the *she1-ΔN126* spindle phenotype is complemented by inactivation of Ipl1/Aurora B or by overexpression of *she1-ΔN126*. The latter shows that premature loading of Ipl1/Aurora B in metaphase inhibits the spindle stabilization effect of *she1-ΔN126*. Furthermore, we show that this novel activity of She1 is negatively regulated by Ipl1/CPC translocation during anaphase.

Recent work suggested that, during the establishment of a functional metaphase spindle midzone (Hepperla et al., 2014), passive cross-linking proteins are required to maintain proper bundling within the spindle once ipMTs are aligned along the spindle axis by kinesin-14 motors. Our results here indicate that She1 might be the sought-after cross-linker that can provide the required maintenance of spindle stability during metaphase.

How She1 prevents premature loading of Ipl1/CPC remains an open question at this point. Our data indicate that the N-terminal domain containing the novel PISH1 and 2 motifs is required (Fig. 5 D). Previous work and our study here show that She1 interacts with Sli15 (Fig. S4 D; Wong et al., 2007). One possibility is that She1 could influence Cdk1- or Ipl1-mediated phosphorylation of Sli15. At least 33 phosphorylation sites have been identified in Sli15 by mass spectrometry (Zimniak et al., 2012; Makrantoni et al., 2014). We speculate that She1’s interaction with Sli15 might increase the extent or the efficiency of its phosphorylation, thereby limiting Ipl1/CPC binding to the spindle in metaphase (Pereira and Schiebel, 2003; Nakajima et al., 2011). A nonspindle-associated pool of She1 could mediate this effect, as *she1-N212*, lacking the MT-binding activity, was able to prevent premature loading of Ipl1 (Fig. 5 D and Fig. S3 G). Alternatively, the interaction between She1 and Sli15 could indirectly (through Sli15’s interaction with Ipl1 in CPC) enhance Cdk1-mediated phosphorylation of Ipl1, which can also negatively regulate CPC–spindle association in metaphase (Zimniak et al., 2012). These possibilities, which are not mutually exclusive, can be experimentally tested in future studies through in vitro kinase assays with purified proteins.

An important conclusion of our study is that She1 activity insures the spindle against possible structural damages during spindle positioning in metaphase. It seems fitting that the protein that regulates the cortical dynein pulling force and directs spindle movement toward the bud compartment (Markus et al., 2012b) should be so intimately involved in maintaining spindle stability when the spindle is pulled through the bud neck. We speculate that similar coordination of spindle stability with spindle positioning may also be required in other cellular contexts where large spindle movements occur, such as during oocyte maturation in mice and *Caenorhabditis elegans*, where the meiotic spindle undergoes a 90° rotation from a parallel orientation at the cortex to a perpendicular orientation to facilitate extrusion of chromosomes into polar bodies (Ellefson and McNally, 2009, 2011), or during asymmetric division of *Drosophila melanogaster* neuroblasts, where the mitotic spindle undergoes a 90° rotation at metaphase to a position perpendicular to the plane of the epidermoblasts to give rise to two daughter cells of different sizes and fates (Kaltschmidt et al., 2000). Based on measurements for *C. elegans* meiotic spindles, Crowder et al. (2015) estimated that an ellipsoid metaphase spindle undergoing a 90° rotation would encounter a significant amount of frictional drag and elastic resistance from the cytoplasm (Crowder et al., 2015). To resist deformation, the rotating spindle would presumably need to be rigid and the two poles be structurally connected to each other so that the spindle axis is pivoted properly. Intriguingly, meiotic spindle rotation in *C. elegans* is also mediated by a dynein-dependent cortical pulling mechanism (Crowder et al., 2015), with cortically anchored dynein reeling in one spindle pole to the cortex via connecting astral MTs (analogous to spindle positioning in budding yeast). Given the high conservation of dynein function from fungi to animals, it is possible that a functional She1 homologue exists in this system (and/or other metazoans) but has eluded identification by sequence-based approaches. It will be interesting to discover whether such a functional homologue could regulate the rotation of the meiotic spindle (or mitotic spindle, as in *Drosophila* neuroblasts) while ensuring the structural integrity of the spindle during rotation.

In addition to controlling dynein activity (Woodruff et al., 2009; Markus et al., 2012b) and maintaining spindle stability during metaphase, She1 has been reported to play a role in the spindle disassembly network that regulates timely spindle disassembly during telophase (Woodruff et al., 2010). In the latter process, She1 appears to act as a spindle destabilizer to promote spindle disassembly, considering that anaphase spindles often rely on cytokinesis to initiate spindle disassembly in the *she1Δ* background (Woodruff et al., 2010). Somewhat paradoxically, this function of She1 is also under the control of Ipl1, as mutating the five Ipl1 phosphorylation sites in She1 resulted in a delayed spindle disassembly phenotype similar to loss of She1 (Woodruff et al., 2010). How does She1 switch from a stabilizer to a destabilizer role, maintaining spindle stability in metaphase and then facilitating its destruction in late anaphase? We reason that She1 probably does not affect MT dynamics in the late anaphase spindle, considering that ipMTs only grow and shrink at the spindle midzone (Maddox et al., 2000) and that She1 does not show strong localization to the midzone (Fig. 6 G; Woodruff et al., 2009; Pigula et al., 2014). We can also exclude regulation of Ipl1 localization by She1 as a possible mechanism in the late anaphase spindle because Ipl1-3GFP decoration of the midzone (Fig. S4 C) as well as removal of Bim1-3GFP from this location (Woodruff et al., 2010) was not detectably affected in *she1Δ* cells. We speculate that an unknown spindle-stabilizing factor might be recruited to the anaphase spindle when She1 is absent, causing spindle disassembly delays. Alternatively, because She1 binds and diffuses along MTs in vitro (Markus et al., 2012b) and localizes along the length and to regions near the spindle poles of the anaphase spindle (Woodruff et al., 2009; Pigula et al., 2014), it is conceivable that She1 prevents the binding of a spindle-destabilizing factor (e.g., an activator of Ipl1) along the ipMTs to promote its focusing to the spindle midzone (e.g., to activate the midzone-localized Ipl1), analogous to the manner by which Glc7/PP1 phosphatase was proposed to drive focusing of Ipl1 toward the spindle midzone (Nakajima et al., 2011). In support of this idea, recent findings by Pigula et al. (2014) showed that defective clearing of She1 from the midzone is associated with spindle disassembly delays in mutants of the environmental stress-sensing HOG pathway (Pigula et al., 2014). Hog1 phosphorylation of She1 is important for its role in spindle disassembly, and intriguingly, the two Hog1 phosphorylation sites in She1 (Thr22 and Thr117; Pigula et al., 2014) are located near and within the N-terminal Ipl1-regulating PISH1 and 2 motifs. Whether Hog1 and/or Ipl1 kinases combinatorially regulate She1 activities, switching its role from a spindle stabilizer (during metaphase) to a spindle destabilizer (during anaphase), will be an important question for future investigations.

## Materials and methods

### Media and strain construction

All *S. cerevisiae* strains used in this study are listed in Table S1 and were originally derived from the genetic background of WT strain YWL36 or YWL37 (Vorvis et al., 2008). DNA was transformed into *S. cerevisiae* cells using a lithium acetate procedure (Gietz et al., 1992; Knop et al., 1999). Transformants were clonally purified by streaking to individual colonies on selective media. Proper tagging or disruption was confirmed by colony PCR and/or sequencing of the genomic locus. At least two independent transformants were chosen from each tagging or disruption procedure for subsequent experiments. Yeast synthetic-defined (SD) media were obtained from Sunrise Science Products. Yeast genomic DNA isolation kit was obtained from Zymo Research Corporation.

To construct plasmids for conditional shutdown of *CDC20* expression, a SacI–NotI PCR product containing a *MET3* promoter sequence was amplified and cloned into SacI- and NotI-digested pRS303 and pRS304, *HIS3*- and *TRP1*-containing vectors (Sikorski and Hieter, 1989), respectively, resulting in pRS303-Met3p and pRS304-Met3p. We next amplified the HIS3-Met3p or TRP1-Met3p cassette with flanking 60 bp targeting the *CDC20* promoter. The PCR product was gel purified and transformed into WT or *she1Δ* strains, generating YWL5219 and YWL4985.

To generate C-terminal *she1* truncation alleles at the genomic locus, we amplified a KanMX6 or 3HA-His3MX6 PCR product from *pFA6a-kanMX6* or *pFA6a-3HA-His3MX6* (Longtine et al., 1998) with flanking 60 bp of homologous sequence targeting the chosen regions to be deleted using the primer pairs listed in Table S2. The PCR product was gel purified and transformed into YWL4736 to yield YWL4745 (*she1-N89* expressing 1–89 aa), YWL4746 (*she1-N126* expressing 1–126 aa), and YWL4748 (*she1-N212* expressing 1–212 aa). To generate N-terminal truncation alleles, we first cloned a SacI–NotI PCR product containing the *SHE1* promoter (1,481 bp upstream of the start codon) amplified from genomic DNA and ligated into SacI- and NotI-digested pRS303 and pRS304, resulting in pRS303-*She1p* and pRS304-*She1p*. A NotI–SmaI PCR product containing a GlyAlaGlyAla linker followed by 90–338 aa (*she1-ΔN89* allele), 127–338 aa (*she1-ΔN126* allele), or 213–338 aa (*she1-ΔN212* allele) was amplified from genomic DNA and ligated into NotI- and SmaI-digested pRS303-*She1p* and pRS304-*She1p*. The resulting tagging plasmids contained the native *SHE1* promoter followed by the GlyAlaGlyAla linker and *she1-ΔN89*, *she1-ΔN126*, or *she1-ΔN212* coding sequence. To facilitate targeting into the native *SHE1* locus and to avoid spurious unwanted recombination events, we linearized the tagging plasmid with PmeI, which cuts within the *SHE1* promoter sequence, and transformed into *she1Δ* cells yielding YWL4829 or YWL4987 (expressing *she1-ΔN89*), YWL4831 or YWL4989 (expressing *she1-ΔN126*), and YWL4833 or YWL4991 (expressing *she1-ΔN212*). Stable His^+^ or Trp^+^ transformants were selected and confirmed by colony PCR.

To construct a tagging vector for N-terminal labeling of She1 with mRuby2 or EGFP at the genomic locus, we first amplified a NotI–SpeI PCR product containing mRuby2 or EGFP amplified from pcDNA3-mRuby2 (plasmid 40260; Addgene) or pKT0128 (Sheff and Thorn, 2004) and stitched it together with a SpeI–SmaI PCR product containing a GlyAlaGlyAla linker followed by the *SHE1* coding sequence amplified from genomic DNA. Next, the stitched mRuby2-linker-She1 or EGFP-linker-She1 PCR product was digested with NotI and SmaI and ligated into the similarly digested pRS303-*She1p* previously described in this paper, yielding pRS303-*She1p*-mRuby2-linker-She1 or pRS303-*She1p*-EGFP-linker-She1. The resulting tagging vector contained the native *SHE1* promoter followed by mRuby2 or EGFP fused in frame to the GlyAlaGlyAla linker and the *SHE1* coding sequence. The tagging vector was linearized with PmeI within the *SHE1* promoter sequence and transformed into a *she1Δ::URA3* strain (Markus et al., 2012b). Stable His^+^ transformants were selected and confirmed by colony PCR.

To localize EGFP-tagged She1 phosphomutants, *SHE1_5A_* and *SHE1_5D_* sequences were amplified from the plasmids pBJ77-She1_5A_ and pBJ77-She1_5D_ (T14A/D, S165A/D, S269A/D, T280A/D, and S325A/D; Markus et al., 2012b), stitched together N-terminally with a PCR product containing EGFP, and ligated into a NotI- and SmaI-digested pRS303-*She1p*-EGFP-linker-She1, generating tagging vectors pRS303-*She1p–*EGFP-linker-She1_5A_ and pRS303-*She1p*-EGFP-linker-She1_5D_. The tagging vectors were confirmed by DNA sequencing, linearized with PmeI within the *SHE1* promoter sequence, and transformed into a *she1Δ::URA3* strain (Markus et al., 2012b). Stable His^+^ transformants were selected and confirmed by colony PCR.

To localize Ipl1, we constructed a tagging vector designed to integrate three tandem copies of GFP at the 3′ end of the *IPL1* genomic locus. In brief, a BamHI–BglII fragment containing the 3′ coding sequence of *IPL1* (723–1,104 bp) and a GlyAlaGlyAlaGlyAla linker were cloned into BamHI-digested pBS-3×GFP-TRP1 (Lee et al., 2003). The resulting plasmid, pBS-Ipl1-3×GFP-TRP1, contained a fragment of *IPL1* sequence fused in frame to the triple GlyAla linker and triple GFP. WT cells were transformed with pBS-Ipl1-3×GFP-TRP1–integrating vector linearized by EcoRV. Stable Trp^+^ transformants were selected and then screened for proper targeting by colony PCR.

For Nuf2 localization, we constructed a tagging vector to integrate mCherry at the 3′ end of the *NUF2* genomic locus. In brief, a ClaI–ClaI fragment containing the 3′ coding sequence of *NUF2* (222–1,353 bp) and a GlyAlaGlyAlaGlyAla linker were cloned into ClaI-digested pHPH-mCherry, a plasmid containing mCherry cloned into pAG32 (Goldstein and McCusker, 1999). The resulting plasmid, pHPH-Nuf2-mCherry, contained a fragment of *NUF2* sequence fused in frame to the triple GlyAla linker and mCherry. The vector was linearized by AfeI and transformed into YWL4985 and YWL4989 to generate YWL5073 and YWL5071. Stable HPH-resistant transformants were selected and then screened for proper targeting by colony PCR.

To label MTs, strains were transformed with BsaBI-digested p*HIS3p:mRuby2-TUB1+3*′*UTR::LEU2* (Markus et al., 2015), undigested *GFP-TUB1::LEU2* (Song and Lee, 2001), or HindIII-digested p*HIS3p:mCherry-TUB1::LEU2*. The latter plasmid was constructed by subcloning a NotI–KpnI fragment containing *HIS3p:mCherry-TUB1* from pAK011 (Khmelinskii et al., 2007) into NotI- and KpnI-digested pRS305, a *LEU2*-containing vector (Sikorski and Hieter, 1989). Leu^+^ transformants were selected and screened by fluorescence microscopy. To replace the endogenous *SHE1* or *CDC20* promoter with *GAL1*p or *GAL1p:3HA* (*GAL1*p with an N-terminal 3HA tag), we performed one-step PCR-mediated transformations using primer pairs listed in Table S2 and the plasmid *pFA6a-kanMX6-PGAL1* or *pFA6a-kanMX6-PGAL1-3HA* as PCR template (Longtine et al., 1998).

### Metaphase block and spotted growth assays

To arrest *MET3p-CDC20* cells in metaphase, overnight cultures grown at 30°C in SD media lacking methionine were diluted into fresh SD media supplemented with 2 mM methionine and then incubated for 5 h at 30°C before imaging. To arrest *GAL1p-CDC20* cells in metaphase, overnight cultures grown at 30°C in SGR media containing 2% galactose and 2% raffinose were diluted into fresh SD media containing 2% glucose (repressing condition) and then incubated for 5 h at 30°C before imaging. In our hands, >90% of *MET3-CDC20* or *GAL1-CDC20* cells were in metaphase after 5 h of arrest, based on bud size and spindle length. To induce the *GAL1*p, overnight cultures grown at 30°C in SD media containing 2% glucose were harvested, washed with water twice, diluted into fresh SGR media containing 2% galactose and 2% raffinose (inducing condition) or fresh SD media containing 2% glucose (noninducing condition), and incubated for 5 h at 30°C before imaging.

To assay for function of chromosomally tagged *IPL1-3GFP*, strains were cultured to mid-log phase in rich yeast extract peptone dextrose (YPD) media, and then 10-fold serial dilutions were spotted on YPD plates and grown for 2 d at 30, 34, and 37°C. To assay for the sensitivity of *IPL1-as5-3GFP* to the ATP analogue 1-NA-PP1 (Tocris Bioscience), serial dilutions of strains were spotted on YPD plates supplemented with or without 50 µM 1-NA-PP1 and grown for 2 d at 30°C.

### Live-cell microscopy and FRAP

For wide-field microscopy, we mounted cells on an agarose pad (1.5%) containing nonfluorescent SD media and captured fluorescence images using either a 1.45 NA 100× objective on an upright microscope (80i; Nikon) equipped with a cooled electron-multiplying charged-coupled device (EMCCD) camera (Cascade II; Photometrics) or a 1.49 NA 100× objective on an inverted microscope system (TiE; Nikon; Marine Biological Laboratory) equipped with a laser launch (405/445/488/514/561/640 nm; LU-NV; Nikon), an adapter (TuCam; Andor), and two EMCCD cameras (Ultra 897; iXon) for simultaneous two-color imaging. Both microscope systems were controlled by NIS-Element software (Nikon). Images were collected at ambient temperature without saturating the camera’s pixels. To minimize phototoxicity to cells and photobleaching during time-lapse imaging of spindle phenotypes in metaphase block, we acquired frames at 30-s intervals and with three optical sections spaced 0.5 µm apart (1 µm in thickness). For imaging of Ipl1-3GFP loading onto the spindle, we acquired frames at 90-s intervals and with five optical sections spaced 0.5 µm apart (2 µm in thickness).

For FRAP analysis of spindle MTs, *MET3-CDC20 GFP-TUB1* cells (WT or *she1Δ* background) were arrested in metaphase by growing in methionine-containing SD media for 3 h before mounting on an agarose pad. FRAP was conducted using a 60× 1.4 NA objective on a confocal microscope (Nikon A1R equipped with a LU-NB laser launch system) housed in the Institute for Applied Life Sciences Nikon Center of Excellence microscopy facility at the University of Massachusetts, Amherst. The pinhole size was set to 0.7 airy units. After photobleaching, images were acquired every 60 s as a stack of five optical sections spaced 0.5 µm apart at room temperature. Measurements of FRAP were made on a maximum-intensity projection of Z series for each time point using established methods (Maddox et al., 2000). In brief, a 10 × 10–pixel square was placed over the bleached area (spindle midpoint or spindle half) or a cytoplasmic reference site (for subtracting background), and integrated intensity was measured three times independently for each dataset. To correct for photobleaching that takes place during image acquisition, we first measured the integrated intensity of unirradiated spindles (eight WT and six *she1Δ* spindles) observed in the same field using the same 10 × 10–pixel square and calculated a normalized photobleaching curve by fitting the intensity values to an exponential decay equation. Next, the integrated intensity of irradiated spindles was divided by the normalized photobleaching curve for each time point. Corrected fluorescence was plotted versus time and fitted with the exponential function A(1 − e^−kt^), where A is the amplitude and k is the recovery rate constant. The *t*_1/2_ for fluorescence recovery was calculated as ln(2)/k. To display the FRAP curves, corrected fluorescence intensities were converted to a fraction of the prebleach fluorescence and normalized to a starting value of 1 (Nakajima et al., 2011).

### Image analysis

To quantify hyperactivity of cortical dynein, we scored for large persistent spindle displacement (>4 µm) during a 10-min video in cells arrested with hydroxyurea or in cells arrested at metaphase by depletion of Cdc20. The ImageJ line tool (National Institutes of Health) was used to measure spindle displacement. Enhancement of cortical dynein activity was also measured by scoring for astral MT detachment from the spindle pole body (Markus et al., 2011; Bergman et al., 2012).

To measure the total fluorescence intensity of tagged proteins (Cin8-3GFP, Ase1-3GFP, Bim1-3mCherry, Ipl1-3GFP, EGFP-She1_WT_, EGFP-She1_5A_, and EGFP-She1_5D_) on the metaphase-blocked spindles, we used the restore selection tool in ImageJ to determine the position of the metaphase spindle based on images of mCherry-Tub1, GFP-Tub1, or mRuby2-Tub1. Using the line tool in ImageJ, we measured the total fluorescence intensity of the tagged protein along the spindle from the maximum intensity projection of Z-stack images. To subtract the background intensity from each measurement, we moved the line tool from the spindle to a nearby cytoplasmic area within the same cell.

To quantify premature loading of Ipl1-3GFP on metaphase spindles arrested by Cdc20 depletion, we quantified the intensity of Ipl1-3GFP fluorescence on the spindle divided by the total intensity of Ipl1-3GFP fluorescence in the nucleus using two-color images of Ipl1-3GFP and mCherry-Tub1. First, we used the restore selection tool in ImageJ to determine the position of the spindle in the Ipl1-3GFP image from the mCherry-Tub1 image. Next, using the line and circle drawing tools in ImageJ, we measured the Ipl1-3GFP intensity along the spindle and within the presumed nucleus (delimited by the spindle pole bodies), respectively, from a maximum intensity projection of Z-stack images. The mean ratio (intensity on the spindle to total intensity in the nucleus) for diffusive nuclear Ipl1-3GFP localization in WT cells was 0.37 ± 0.1 (mean ± standard deviation, *n* = 107). We thus defined cells displaying ratios >0.5 (i.e., one standard deviation above the mean in WT) as having an enhanced Ipl1 phenotype.

To calculate the correlation coefficient between the localization of Ipl1-3GFP and mRuby2-She1 or Sli15-mRuby2, the position of the anaphase spindle was determined and selected based on images of Ipl1-3GFP and then cropped to minimize nonspindle regions. Next, using the threshold tool, we converted the fluorescence intensities along the spindle into binary signals, and, using the Coloc 2 tool, we calculated the Pearson’s correlation coefficient between Ipl1-3GFP and mRuby2-She1 or Sli15-mRuby2 on each spindle.

### Protein purifications

To construct the expression plasmid for HALO-tagged She1-FL, a PCR product containing the coding sequence of She1-FL (without the stop codon) was amplified from yeast genomic DNA using primer pairs listed in Table S2, digested with AvrII, and cloned into NheI- and AvrII-digested pGEX-KG-SUMO-PAC1-HALO vector (bWL579, generated by subcloning Smt3 and Pac1 coding sequences into pGEX-KG), resulting in pGEX-KG-SUMO-SHE1-HALO. Plasmids for HALO-tagged She1-N (aa 1–212) and She1-C (aa 194–338) were constructed similarly. To generate the expression plasmid for HALO-tagged She1_5D_, we used the multisite-directed mutagenesis kit (QuikChange Lightning; Agilent Technologies), yielding pGEX-KG-SUMO-SHE1_5D_-HALO. To construct the expression plasmid for HALO-tagged Sli15, we first amplified a BamHI–EcoRI PCR product containing the SUMO sequence and cloned it into BamHI- and EcoRI-digested pGEX-6P-1 plasmid (GE Healthcare), resulting in pGEX-6P-1-SUMO. We next introduced a NotI–PspOMI PCR product containing the HALO sequence amplified from bWL579 into NotI-digested pGEX-6P-1-SUMO, generating pGEX-6P-1-SUMO-HALO. A SacI–XhoI PCR product containing the coding sequence of full-length Sli15 (without the stop codon) was amplified from yeast genomic DNA and cloned into SacI- and XhoI-digested pGEX-6P-1-SUMO-HALO, resulting in pGEX-6P-1-SUMO-SLI15-HALO.

To purify HALO-tagged She1-FL, She1-N, She1-C, or Sli15, T7 Express *lysY/I^q^* cells (New England Biolabs) carrying pGEX-KG-SUMO-SHE1-HALO, pGEX-KG-SUMO-SHE1-N-HALO, pGEX-KG-SUMO-SHE1-C-HALO, pGEX-KG-SUMO-SHE1_5D_-HALO, or pGEX-6P-1-SUMO-SLI15-HALO were induced with 0.1 mM IPTG at 16°C for 18 h. Cells were harvested and sonicated in ice-cold lysis buffer (30 mM HEPES, pH 7.4, 0.1 mM EGTA, 100 mM potassium acetate, 2 mM magnesium acetate, 1 mM DTT, and 0.1% Triton X-100) supplemented with a protease inhibitor tablet (Roche Applied Science). After centrifugation, the supernatant was added to GST-bind resin (Novagen) and incubated at 4°C for 1 h with gentle rotation followed by extensive wash with washing buffer (lysis buffer supplemented with 200 mM KCl) and MAP-binding buffer (10 mM HEPES, pH 7.4, 150 mM KCl, 1 mM DTT, 2 mM MgCl_2_, 1 mM EGTA, and 5% glycerol). To label with fluorescent probes, bound proteins on GST-bind resin were incubated with HaloTag TMR or Alexa Fluor 488 ligand in MAP buffer at room temperature for 20 min. Purified HALO-tagged fusion proteins were released from GST-bind resin by Ulp1 digestion in MAP buffer at 4°C overnight. Protein concentration was determined by Bradford protein assay (Bio-Rad).

### MT-binding and cross-linking assays

To prepare Taxol-stabilized MTs, we polymerized 40 µM bovine tubulin (Cytoskeleton) in PEM buffer (80 mM PIPES, pH 6.9, 1 mM MgCl_2_, 1 mM EGTA, and 1 mM GTP) at 37°C for 20 min followed by incubation for another 20 min with 40 µM Taxol (Cytoskeleton). The MTs were pelleted (in a TLA100 rotor at 48,000 rpm and 25°C for 10 min) and resuspended to a final concentration of 40 µM tubulin dimer in PEM buffer containing 40 µM Taxol. HiLyte 488–labeled MTs were prepared similarly by mixing with unlabeled tubulin at a 13:1 ratio.

For the MT-binding assay, the following solutions were introduced sequentially into a flow chamber constructed using a slide and a silanized coverslip attached with double-sided adhesive tape: (a) 20 µg/ml antitubulin antibody (1F4E3; GenScript), (b) blocking buffer containing 1% Pluronic F-127 and 1 mg/ml κ-casein, and (c) a mixture of 60 nM Taxol-stabilized HiLyte 488–labeled MTs and 1 µM purified TMR-labeled She1-FL, She1-N, or She1-C protein preincubated at room temperature for 20 min in MAP-binding buffer containing 150 mM KCl and 20 µM Taxol. After a 15-min incubation at room temperature, the chamber was washed twice with MAP-binding buffer and then imaged on a wide-field fluorescence microscope (80i; Nikon) equipped with a 1.45 NA 100× objective, a motorized filter cube turret, and an EMCCD camera (Cascade II). We used sputtered/ET filter cube sets (Chroma) for imaging HiLyte 488 (49002) and TMR (49008) fluorescence. The same microscope settings were used for capturing all images. Bound TMR fluorescence on the MT was measured using the segmented line tool in ImageJ. MT cross-linking assay was performed similarly except that 60 nM Taxol-stabilized, HiLyte 488–labeled MTs were preincubated either alone or with 1 µM purified TMR-labeled She1-FL, She1-N, or She1-C at room temperature for 20 min in MAP buffer containing 15 mM KCl and 20 µM Taxol before adding to the flow chamber.

For the bulk MT cross-linking assay, 10 µM Taxol-stabilized MTs were mixed with or without 8 µM She1-C protein in MAP buffer containing 15, 50, 100, or 150 mM KCl. After 20-min incubation at room temperature, the absorbance at 340 nm was measured using a spectrophotometer (DU730; Beckman Coulter). Three independent replicates were performed for each salt concentration.

For the low-speed microfuge-based pelleting assay, varying concentrations of Taxol-stabilized MTs in MAP buffer containing 50 mM KCl were incubated for 20 min with 5 µM purified TMR-labeled She1-C at room temperature. Next, reactions were centrifuged for 10 min at room temperature in a microfuge (model 5424; Eppendorf) at 13,000 rpm (15,871 *g*). Pellet fractions were subjected to SDS-PAGE, and fluorescence intensities of She1-C–TMR bands were imaged using a gel documentation system (G:Box Chemi HR16; Syngene) equipped with a 16-bit charge-coupled device camera (ICX285AL; Sony), an overhead 275–375-nm UV illumination, and an emission filter (SG03; Syngene). To measure band intensities, rectangular regions of equal size were drawn around each band (using ImageJ), and the background-corrected integrated intensity was recorded. The fraction of She1-C–TMR that copelleted with MTs was obtained by dividing the integrated intensity of the pellet fraction by the intensity of the input. Because MTs were sedimented at low speed, the pellet fraction represents MTs that were cross-linked by She1-C–TMR. Thus, to determine the stoichiometry of She1-C–TMR and tubulin in the cross-linked MTs, we fitted the data with a quadratic equation for the fraction of pelleted She1-C–TMR, f = *A*[*Q* − (*Q*^2^ − 4*C*_MT_*C*_She1-C_)^1/2^]/[2*C*_She1-C_], where *Q* = *K*_d_ + *C*_MT_ + *C*_She1-C_, with *K*_d_ being the apparent dissociation constant of She1-C with the cross-linked MTs, *C*_MT_ and *C*_She1-C_ being the total concentrations of tubulin and She1-C–TMR in the experiment, respectively, and *A* being the amplitude of the reaction. The amount of MTs that were cross-linked by 5 µM She1-C–TMR was estimated from the saturation point of the fitted curve, determined as the intercept between the plateau and the linear regression of the initial slope.

### EM and analytical ultracentrifugation

Negatively stained samples for EM were prepared as described in Booth et al. (2011). In brief, aliquots of purified She1-FL were blotted onto freshly glow-discharged carbon-coated copper grids (CF400-CU; Electron Microscopy Science) and incubated at room temperature for 30 s. The grids were then briefly washed with sterile double-distilled water and stained with 0.75% uranyl formate for 30 s. Images from air-dried grids were captured on a transmission electron microscope (JEOL 100CXII; housed in the Center for Microscopy and Imaging at Smith College, Northampton, MA) operating at 100 kV with magnifications of 80,000× or 100,000×. Length measurements of She1-FL particles in electron micrographs were performed using ImageJ software.

Sedimentation velocity was performed at 20°C using an An-50 Ti rotor in an ultracentrifuge (Optima XLI; Beckman Coulter) equipped with a fluorescence detection system (AVIV Biomedical) housed in the Institute for Applied Life Sciences Biophysical Characterization Core Facility at the University of Massachusetts, Amherst. We loaded purified Alexa Fluor 488–labeled She1-FL and She1-N in buffer containing 10 mM Tris, pH 8, 150 mM KCl, 1 mM DTT, and 0.1 mg/ml BSA into two-sector epon centerpieces and centrifuged them at 20,000 or 40,000 rpm. Fluorescence was detected and monitored with 488-nm excitation and 505–565-nm emission. We collected scans continuously with no delay and used the SEDFIT software (v15.01) to determine the distribution of sedimentation coefficients (Zhao et al., 2013).

### Western blotting and pull-down assay

For immunoblotting, overnight yeast cultures were harvested and washed once with sterile water. An equal amount of pelleted cells was resuspended in 450 µl of ice-cold lysis buffer containing 20 mM Tris, pH 7.5, 1.5% Triton X-100, 150 mM NaCl, and 1 mM EDTA supplemented with protease inhibitor tablet (Roche Applied Science) and lysed by bead beating six times for 1 min, with 2 min on ice between each beating. Equal loading of crude lysate was analyzed by SDS-PAGE followed by Western blotting. Mouse anti–c-Myc antibody (A00704; Genscript) and HRP-conjugated goat anti–mouse antibody (A00160; Genscript) were used at 1:5,000 and 1:10,000 dilutions, respectively.

To prepare GST-Sli15 beads for pull-down assay, 1 liter of T7 Express *lysY/I^q^* cells carrying pGEX-6P-1-SUMO-Sli15-HALO was harvested, lysed, and clarified as described by Tang et al. (2012). The supernatant was added to 0.6 ml GST-bind resin (Novagen), incubated at 4°C for 45 min with gentle rocking, and washed three times with washing buffer (30 mM HEPES, pH 7.4, 0.1 mM EGTA, 100 mM potassium acetate, 2 mM magnesium acetate, 1 mM DTT, 0.1% Triton X-100, and 200 mM KCl) and two times with TEV buffer (10 mM Tris, pH 8.0, 150 mM KCl, 10% glycerol, 1 mM DTT, and 0.1% Triton X-100). The final washed beads were resuspended in 0.2 ml of yeast lysis buffer (20 mM Tris, pH 7.5, 100 mM NaCl, 1 mM DTT, 0.1% Triton X-100, and 1 mM EDTA) supplemented with protease inhibitor tablet (Roche Applied Science). Equal amounts of yeast extract expressing either full-length *SHE1-13myc* or *she1-ΔN126-13myc* were prepared and incubated with an equal amount of GST-Sli15 immobilized on glutathione beads at 4°C for 45 min with gentle rocking. Resins were washed two times with yeast lysis buffer, and bound proteins were eluted with 1× gel sample buffer and analyzed by SDS-PAGE followed by Western blotting.

### Statistical methods

Statistical significance was determined by comparing datasets with different numbers of trials or independent data points using a Student's *t* test on unpaired data with unequal variance. Data distribution was assumed to be normal, but this was not formally tested. Probability values and number of trials are given in the figure captions where appropriate.

### Online supplemental material

Supplemental data of this article include seven videos, four additional figures, and two tables showing sequence analysis of fungal She1 homologues (Fig. S1); function, immunoblotting, and localization analyses of She1 (Fig. S2, A–C); characterizations of spindle and chromosome segregation defects in *she1* mutants (Fig. S2, D–H; Fig. S3, A–D; and Videos 1–5); sedimentation analysis of She1-N (Fig. S3, E and F); localization of mRuby2-tagged She1 constructs (Fig. S3 G); timing of Ipl1-3GFP spindle localization (Fig. S4, A–C; and Videos 6 and 7); GST-Sli15 pull-down assay of She1 constructs (Fig. S4 D); rescue of the bent spindle phenotype by overexpression of *she1* alleles (Fig. S4 E); and yeast strains and primers used in this study (Tables S1 and S2).
